# Epidemiological study of sausage in Algeria: Prevalence, quality assessment, and antibiotic resistance of *Staphylococcus aureus* isolates and the risk factors associated with consumer habits affecting foodborne poisoning

**DOI:** 10.14202/vetworld.2019.1240-1250

**Published:** 2019-08-15

**Authors:** Amina Hachemi, Safia Zenia, Mohamed Fatih Denia, Meryem Guessoum, Mohamed Mehdi Hachemi, Khatima Ait-Oudhia

**Affiliations:** 1 Laboratory of Food Hygiene and Quality Insurance System (HASAQ), Higher National Veterinary School, Rue Issad Abbes, Oued Smar, Algiers 16000, Algeria; 2 Research Laboratory Management of Local Animal Resources (GRAAL), Higher National Veterinary School, Rue Issad Abbes, Oued Smar, Algiers 16000, Algeria; 3 Laboratory of Medical Biology, Beraki Road, BP 71, El Harrach, Algiers, Algeria; 4 Municipal Office of Environment, Algiers 16000, Algeria

**Keywords:** consumers, quality assessment, risk factors, sausages, *Staphylococcus aureus*

## Abstract

**Aim:**

The first aim was to assess the quality and determine the prevalence and antibiotic susceptibility of *Staphylococcus aureus* contamination of raw sausage sold in ten municipalities in the Northeast of Algeria. Second, a consumer sausage purchasing survey was designed to investigate potential risk factors that have a significant association with the occurrence of foodborne poisoning among sausage consumers’ behavior and its relationship with independent variables.

**Materials and Methods:**

A total of 230 butcheries from ten departments (Daira) of Algiers with more than 40 municipalities were included randomly in these studies to collect raw sausage samples and to distribute 700 structured questionnaires to meat consumers. Our two studies were conducted at the same time, between June 2016 and April 2018. Sausage samples were taken once per butchery to estimate the prevalence of *S. aureus* contamination and therefore deduct the quality assessment of raw sausage (Merguez) sold in Algiers, Algeria. All isolated strains were tested for their antimicrobial resistance. Furthermore, questionnaires were distributed and used to collect information on various aspects of sausage consumption and foodborne disease. The data collected were analyzed with different statistical approaches, such as the Chi-square test and the odds ratio (OR) univariable logistic model. All the risk factors were analyzed by studying their association with the occurrence of consumers who claimed to have food poisoning after consuming sausage.

**Results:**

The overall prevalence of *S. aureus* contamination from sausages was 25.22% (n=58/230). Over 83.33% of strains showed resistance to at least one of the antibiotics tested. The most important was for tetracycline (58%) followed by fosfomycin (33%), penicillin G (25%), and oxacillin (36%). Moreover, the multiple antibiotic resistance (MAR) index include 20 profiles with MAR >0.2. Out of the 440 meat consumers, 22.16% revealed having food poisoning after sausage consumption. The risk factors recorded were: Consumption outside of home (24.30%, OR=1.769, p=0.040), during the summer season (24.30%, OR=1.159) and during lunch (26.50%, OR=1.421).

**Conclusion:**

Our study highlights a high prevalence of *S. aureus* contamination in Merguez, especially in some departments of Algiers, and the high multidrug resistance of *S. aureus* isolates against tetracycline and oxacillin; thus, *S. aureus* contamination in sausage is considered a potential risk to public health. Therefore, to reduce and prevent the spread of resistant strains, robust management and monitoring of antibiotic use should be established. Therefore, it is necessary to improve the sanitation conditions and education regarding personal hygiene and change certain consumption habits of Algerian consumers to ensure food safety. Finally, it can be concluded that the application of the HACCP system is essential either in butcheries producing sausage and/or slaughterhouses. From this perspective, studies might be performed to characterize *Staphylococcus* spp and *S. aureus* to investigate their virulence factors.

## Introduction

*Staphylococcus* is a genus composed of two groups: Coagulase positive and negative [1]. Of the coagulase-positive Staphylococci group, *Staphylococcus aureus* has been reported as the third most common cause of foodborne diseases around the world [2]. In 2018, the Centers for Disease Control in the United States estimated that 48 million people each year get sick from a foodborne illness, 128,000 are hospitalized, and 3000 dies [3]. This situation is all the more serious in emerging nations, with devastating economic consequences [4]. According to the Algerian Ministry of Health, more than 15,233 cases of food poisoning were recorded between 2016 and 2017, with 16 deaths, of which *S. aureus* was the second leading cause [5]. Furthermore, *S. aureus* has been identified by the World Health Organization as an international concern due to its multidrug resistance [6]. In addition, *S. aureus* can cause mastitis in cows and small ruminants, resulting in the animals being asymptomatic carriers or suffering from respiratory, gastrointestinal, or skin problems [7]. Moreover, the presence of antibiotic-resistant strains has become an emerging zoonotic issue of public health concern [8] and important vehicles for transferring antimicrobial resistance factors to the intestinal tract of consumers [9]. Despite extensive research efforts, many people still suffer from Staphylococcal infections [10]. *S. aureus* is a common pathogen associated with community and nosocomial acquired diseases [11], and a possible treatment remains complicated [12], especially with the appearance of methicillin resistance found in several species of meat-producing animals, including pigs, chickens, and cattle [13], increasing the concern about human exposure to *S. aureus* through the food chain.

The commodities we are interested in are specifically spicy lamb or beef-based raw sausage, a North African specialty called “Merguez.” Merguez is regarded as the most popular variety of meat products widely consumed in Algeria. Due to its nutritional composition, sausage constitutes a rich medium that is very favorable to pathogen growth; most pathogens are inhibited, except *S. aureus* which is able to grow under a wide range of environmental conditions [14]. Studies suggest that generally the number of *S. aureus* required to produce an outbreak of Staphylococcal food poisoning is approximately 10^5^-10^6^ CFU/g or mL [15], and it is associated with nausea, vomiting, diarrhea, and abdominal pain within a few hours after ingestion [16]. As a consequence, research has been conducted to investigate the presence of *S. aureus* in different kind of meats, as described previously [17-27].

Despite the fact that Merguez is a typical product of North Africa, data concerning *S. aureus* detection in sausage are limited to the Moroccan study described by Ed-Dra *et al*. [14] and a handful of studies conducted in Spain [28], Italy [29], Turkey [30], the USA [31], and Saudi Arabia [24]. There is an increased risk for foodborne diseases caused by sausage, but unfortunately, there have not been studies on sausage consumption in Algeria, even though meat product consumption behavior has changed remarkably during the past few years as a direct consequence of the market economy and the search for more quality products and other credence characteristics. Focusing on Merguez, more information is necessary to investigate the importance of this meat product in Algerian society and to report a survey of sausage consumption patterns, attempting to estimate the impact of a possible sausage contamination on the health of Algerian consumers, and to determine the magnitude of the problem and the different risk factors related to sausage consumption.

To the best of our knowledge, the present paper is the first study on sausage in Algeria; as well as on the bacteriological portion and consumer survey. Therefore, the aims of our epidemiological cross-sectional study are the following: (i) To estimate the prevalence and distribution of *S. aureus* contamination in different departments (Daira) of Algeria, (ii) to investigate the quality assessment and the antimicrobial susceptibility of *S. aureus* isolates in artisanal sausage (Merguez), destined for food consumption in ten Daira of Algiers, Algeria, and (iii) to establish the first Algerian epidemiological survey on sausages consumers, a survey seeking to identify sausage consumption habits and to investigate the various risk factors influencing the occurrence of foodborne poisoning among sausage consumers. Our findings will help authorities establish risk management strategies, prevent foodborne outbreaks, and avoid the spread of foodborne pathogens (e.g., *S. aureus*) throughout the food chain further filling in data deficits in this regard.

## Materials and Methods

### Ethical approval

Sausages were taken from butcheries, which did not need contact with animals. The present study did not involve any invasive procedure, and hence, ethical approval is not required.

### Informed consent

Informed consent was obtained from each participants.

### Study area and design of sampling sites

**Figure-1:**
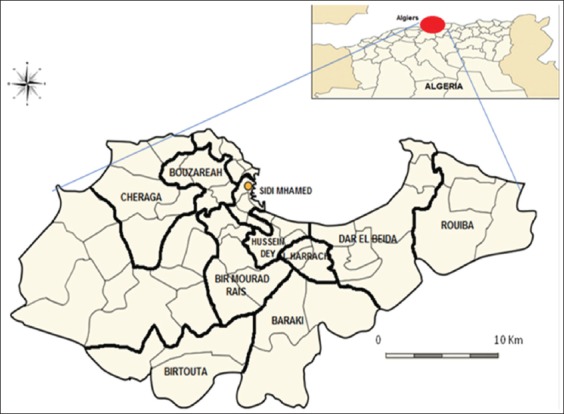
Geographical distribution of the study areas consist of the sampling sites in ten departments (More than 40 municipalities) of Algiers, capital of Algeria (230 butchers sampling). Source: Personalized map (Based on geographic delineation maps provided by Algiers department).

Between June 2016 and April 2018, sausage samples of 230 butcheries were randomly collected from ten (out of 13) departments (Daira) with 43 municipalities located in urban and peri-urban areas of Algiers, the capital of Algeria (Sidi M’hamed, Bir Mourad Rais, Bab El Oued, El Harrach, Hussein Dey, Dar El Beida, Bouzareah, Cheraga, Zeralda, Birtouta, Daira, Beraki, and Rouiba) (Figure-1). The samples at the same butchery were taken once but from different lots, with an average of 23 samples per department (Daira). Merguez samples were identified using the standard ISO 6888-1 [32] and included for further investigation. Samples were obtained aseptically from display cases, and approximately 500 g of the sausage was transported to the laboratory at 4°C in ≤2 h. In no case was the sample frozen. Direct contact with the sample was carried out under strict aseptic conditions [33].

### *S. aureus* isolation and identification

Microbiological isolation and identification were performed according to techniques recommended by the International Organization for Standardization ISO 6888-1:1999 Standard, using Baird-Parker agar with egg yolk-potassium tellurite emulsion plates (BP, Pasteur Institute of Algiers) incubated at 37°C for 24-48 h [22]. Five presumptive *S. aureus*-typical colonies (Typical colonies: Black, surrounded by a clear zone) were subcultured and identified by conventional methods (Gram staining, catalase test, mannitol fermentation, and the ability to coagulate rabbit plasma) [34,35] followed by a latex agglutination test by Pastorex Staph Plus assay (Bio-Rad, Marnes-la-Coquette, France) [12]. The isolates that responded positively to the mentioned tests were considered *S. aureus* strains.

### Quality assessment

Sausage quality was evaluated and interpreted with Regulation (EC) No. 2073/2005 on microbiological criteria for foodstuffs [36] and according to the Algerian Food Codex for assessing the microbiological safety of the *S. aureus* in the artisanal sausage [37]. Poor Merguez quality is attributed as “unsatisfactory quality” with contamination levels exceeding 3.70 log CFU/g. In contrast, good Merguez quality is attributed as Merguez belonging to “satisfactory quality” with *S. aureus* contamination levels ≤2.70 log CFU/g and/or “acceptable quality” with *S. aureus* contamination levels between 2.70 and 3.70 log CFU/g.

### Antimicrobial susceptibility testing

Antimicrobial susceptibility was performed for all the confirmed strains according to the standard disk diffusion method on Müller-Hinton agar against the following panel of 16 antimicrobial agents (charge in µg/disk) (Sirscan, France): Penicillin G (1UI), cefoxitin (30), oxacillin (1), vancomycin (30), teicoplanin (30), erythromycin (15), gentamicin (10), ciprofloxacin (5), fosfomycin (200), rifampicin (30), ofloxacin (5), chloramphenicol (30), tetracycline (30), levofloxacin (5), kanamycin (30) and trimethoprim/sulfamethoxazole (1.25/23.75). Minimum inhibitory concentrations were determined using the E-test method (bioMérieux) [35]. Strains were classified as resistant in accordance with EUCAST [38] for all antibiotics tested, except for oxacillin, which was evaluated according to EUCAST [39]. The multiple antibiotic resistance (MAR) index was calculated.

### Study population

#### Questionnaire survey

A cross-sectional epidemiological survey was carried out from June to November 2018 and included a total of 700 structured questionnaires distributed to Algerian meat consumers from the same butcheries of the municipalities described above (n=230). The questionnaire contained close-ended questions used to collect information on various aspects of sausage consumption and foodborne disease. The questionnaire consisted of structured questions divided into three categories. The first category compromised questions regarding the demographic characteristics of the respondents (age, gender, residence, having children, and purchase location). The second category involved information concerning consumption habits (place, season, and time of sausage consumption) and the third includes storage conditions (length of transport, refrigeration, and freezing of sausages) and their relationship with the occurrence of food poisoning in the respondents following sausage consumption. The questionnaire was pretested, modified, and refined before starting.

### Statistical analysis

The data collected were analyzed in IBM SPSS statistical software version 20.0 (IBM, USA) for Windows with different statistical approaches. Descriptive statistics by the Chi-square test and odds ratio (OR) univariable logistic model were performed to analyze the potential risk factors and their relation to the independent variables. All analyses were carried out at a 95% confidence level with the significance level fixed at p<0.05.

## Results

### *S. aureus* prevalence

**Figure-2:**
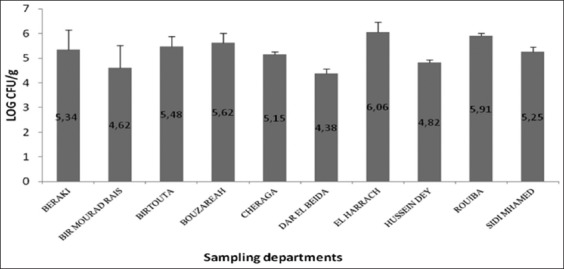
The average values of *Staphylococcus aureus* counted in sausages according to the sampling departments.

Our study was carried out to estimate the prevalence of *S. aureus* among sausages collected from 230 butcheries in 10 departments (Daira) in Algiers. Fifty-eight (230) samples were contaminated with *S. aureus*. Samples taken from the ten departments were analyzed by the Chi-square homogeny test with p=0.025 and determined to be homogeneous. The overall prevalence of 25.22% (IC [19.60-30.80]) was obtained, and the highest prevalence was found in Beraki (68%), Cheraga (44%), and El Harrach (43.75%). The least prevalent samples were recorded from Sidi M’hamed (4.76%), Dar El Beida (5%), and Rouiba (8.70%), with significant differences between the ten Daira, p<0.05 (Table-1). Furthermore, based on quantitative analysis, the overall mean of *S. aureus* contamination was 5.26±0.45 log CFU/g, with IC values between 5.202 and 5.318. The minimum contamination amount in all departments was observed in the Dar El Beida sample, with 4.38±0.00 log CFU/g, while the maximum was recorded in the El Harrach sample, at 6.06±0.2 log CFU/g, as represented in Figure-2.

**Table 1:** Prevalence, contamination level, and quality assessment of *S. aureus* isolated from sausage.

Cities of Algiers	Analyzes butcheries^a^	*S. aureus* contamination in raw sausage					

		Positives butcheries^d^	Prevalence^ab^ (%)	Contamination level^a^ (Log CFU/g)			Quality assessment^a^
	
				Minimum	Maximum	Mean±SD	Poor quality^c^ Nb. (%)
Beraki	25	17	68.00	4.30	6.19	5.34±0.6	17 (86.00)
Bir Mourad Rais	25	6	24.00	3.37	5.41	4.62±0.7	5* (20.00)
Birtouta	25	3	12.00	5.26	5.75	5.48±0.2	3 (12.00)
Bouzareah	25	5	20.00	5.22	5.81	5.62±0.2	5 (20.00)
Cheraga	25	13	52.00	4.04	6.08	5.15±0.8	13 (52.00)
Dar El Beida	20	1	5.00	4.38	4.38	4.38±0.0	1 (5.00)
El Harrach	16	7	43.75	5.72	6.24	6.06±0.2	7 (43.75)
Hussein Dey	25	3	12.00	4.30	5.82	4.82±0.9	3 (12.00)
Rouiba	23	2	8.70	5.29	6.55	5.91±0.9	2 (8.70)
Sidi Mhamed	21	1	4.76	5.25	5.25	5.25±0.0	1 (4.76)
Total	230	58	25.22	4.71	5.75	5.26±0.45	57/230 (24.78)

a Distribution per city;

b Prevalence of contamination;

c Nonsatisfaction quality;

d Butcheries where *S. aureus* was isolated;

* The case of acceptable quality has been removed. *S. aureus*=*Staphylococcus aureus*

### Quality assessment of sausages

Concerning the quality assessment of the sausages analyzed, 24.78% (n=57/230) of the samples represented poor sausage quality (unsatisfactory quality) due to *S. aureus* contamination with IC (19.20-30.60), as presented in Table-1. The worst qualities were recorded in Beraki, Cheraga, and El Harrach with (68%; n=17/25), (52%; n=13/25), and (43.75%; n=07/16), respectively. Dar El Beida (5%; n=01/20), Sidi Mhamed (4.76%; n=01/21), and Rouiba (8.70%; n=02/23) were the departments with the best sausage quality. The analysis showed a significant difference between Daira (p<0.05).

### Antimicrobial susceptibility profile

The resistance patterns of all the *S. aureus* isolates against the tested antibiotics are shown in Table-2. As reported, the results of the antimicrobial susceptibility testing demonstrate *S. aureus* resistance to a panel of 16 antibiotics belonging to different classes, including penicillin G (25%, n=21/84), oxacillin (36%, n=30/84), cefoxitin (05%, n=04/84), erythromycin (23%, n=19/84), ciprofloxacin (13%, n=11/84), ofloxacin (19%, n=16/84), rifampicin (13%, n=11/84), gentamicin (2%, n=02/84), chloramphenicol (10%, n=8/84), trimethoprim/sulfamethoxazole (4%, n=3/84), tetracycline (58%, n=49/84), levofloxacin (14%, n=12/84), and fosfomycin (33%, n=28/84). No resistance was recorded for vancomycin, teicoplanin, or kanamycin. The results show *S. aureus* strains highly resistant to tetracycline, which is the most common antibiotic resistance profile from all department samples, followed by oxacillin and fosfomycin. In contrast, very low resistance was found for gentamycin and trimethoprim/sulfamethoxazole. Furthermore, 15 isolates (17.85%) were susceptible to all tested drugs. A total of 21 *S. aureus* isolates were resistant to only one antimicrobial (25%); 20 (24%) were resistant to two antimicrobials; and 28 (33%) were resistant to three or more antibiotics most commonly used in veterinary medicine. Over 70 (83.33%) of the isolated *S. aureus* strains showed resistance to at least one of the antibiotics tested. Moreover, the analysis of our results showed that the MAR index varies between 0 and 0.63 with 39 different phenotypic profiles (Table-3), including 20 profiles (23 *S. aureus* isolates) with MAR >0.2. Out of the 16 oxacillin-resistant isolates (n=16/84; 19.04%), four (n=4/84; 4.80%) showed additional resistance to cefoxitin and penicillin by antimicrobial susceptibility and were thus identified as methicillin-resistant *S. aureus* (MRSA) strains as described [40]. All MRSA isolates showed resistance to tetracycline and 1, 3, or 5 other antibiotics.

**Table 2:** Antibiotic resistance of *S. aureus* isolated from sausage (n=84).

Antibiotic class	Antibiotics	*S. aureus*, n (%)
B-Lactams	P	21 (25)
	OXA	30 (36)
	FOX	4 (5)
Macrolides	E	19 (23)
Quinolones	CIP	11 (13)
	OFX	16 (19)
	LEV	12 (14)
RAM	RAM	11 (13)
Glycopeptides	VA	0
	TEC	0
Aminoglycosides	CN	2 (2)
	KMN	0
Phenicoles	C	8 (10)
Sulfonamides	SXT	3 (04)
Others	TE	49 (58)
	FOS	28 (33)
ORT		21 (25)
TRT		20 (24)
ThRT		28 (33)

ORT=Resistance to one antibiotic class, TRT=Resistance to two antibiotic class, ThRT=Resistance to three antibiotic class, *S. aureus*=*Staphylococcus aureus, P=Penicillin G, OXA=Oxacillin, FOX=Cefoxitin, E=Erythromycin, CIP=Ciprofloxacin, OFX=Ofloxacin, LEV=Levofloxacin, RAM=Rifampicin, VA=Vancomycin, TEC=Teicoplanin, CN=Gentamycin, KMN=Kanamycin, C=Chloramphenicol, SXT=Sulfamethoxazole/trimethoprim, TE=Tetracycline, FOS=Fosfomycin*

**Table 3:** Resistance profile of *S. aureus* isolated.

Number of antibiotics	Number of isolates	Resistance profile of isolate *S. aureus*	MAR index
0	15	-	0.00
1	4	OXA	0.06
	9	TE	0.06
	3	P	0.06
	3	FOS	0.06
	2	RAM	0.06
2	1	TE-FF	0.13
	1	CIP-C	0.13
	2	P-TE	0.13
	1	E-FOS	0.13
	1	OXA-E	0.13
	1	OXA-FOS	0.13
	1	OXA-OFX	0.13
	1	OXA-RAM	0.13
	1	OXA-TE	0.13
	7	TE-FOS	0.13
3	3	OXA-TE-FOS	0.19
	3	E-TE-FOS	0.19
	1	P-OXA-C	0.19
	1	P-OXA-TE	0.19
	1	OXA-TE-FOS	0.19
4	1	OXA-E-RAM-TE	0.25
	1	P-E-TE-C	0.25
	1	P-OXA-CN-FOS	0.25
	1	OXA-OFX-RAM-TE	0.25
5	1	P-OXA-E-RAM-TE	0.31
	1	P-OXA-OFX-TE-FOS	0.31
	1	P-OXA-FOX-TE-FOS	0.31
	1	P-OXA-E-TE-FOS	0.31
	1	P-CIP-OFX-TE-LEV	0.31
	1	E-CIP-OFX-TE-LEV	0.31
	1	OXA-E-OFX-TE-LEV	0.31
6	2	P-FOX-CIP-OFX-TE-LEV	0.38
	1	OXA-E-CIP-OFX-TE-LEV	0.38
	1	OXA-E-OFX-RAM-TE-FOS	0.38
	1	P-OXA-E-RAM-TE-C	0.38
	1	E-CIP-OFX-TE-FOS-LEV	0.38
7	1	P-E-CIP-OFX-RAM-TE-LEV	0.44
8	1	OXA-E-CIP-OFX-TE-LEV-C-SXT	0.50
9	1	P-OXA-FOX-OFX-CN-TE-FOS-LEV-C	0.56
10	2	P-OXA-E-CIP-OFX-RAM-TE-LEV-C-SXT	0.63

MAR=Multiple antibiotic resistance, OXA=Oxacillin, TE=Tetracycline, E=Erythromycin, P=Penicillin G, FOS=Fosfomycin, RAM=Rifampicin, CIP=Ciprofloxacin, OFX=Ofloxacin, CN=Gentamycin, LEV=Levofloxacin, FOX=Cefoxitin, SXT=Sulfamethoxazole/trimethoprim, C=Chloramphenicol

### Risk factor analysis

Our findings demonstrated the highest consumption frequency of sausage (n=379/440, 86.14%) among meat consumers who responded to our questionnaire (440/700). Out of 379 sausage consumers, 84 (22.16%) claimed to have food poisoning after consuming sausage. Our analysis identified three categories that underline 11 risk factors among the 14 related to sausage consumer habits with an OR >1. The distribution of the risk factors was as follows: Demographic characteristics, consumption habits, and storage conditions. The OR analysis of the assumed risk factors is summarized in Table-4. However, only two risk factors were found to be statistically significant, with p=0.04 and p=0.025 for consumers eating outside of the house and in the Birtouta department, respectively.

**Table 4:** Risk factors analysis.

Risk factors	Number of sick consumers (%)	p-value	OR	CI

1. Demographic characteristics				
Age				
18-40 years	74/328 (22.60)	0.637	1.194	0.571-2.499
41-75 years	10/51 (19.60)			
Gender				
Males	28/109 (25.70)	0.294	1.321	0.785-2.224
Females	56/270 (20.70)			
Habitat				
With family	73/310 (23.50)	0.169	1.624	0.810-3.257
Alone	11/69 (15.90)			
Having children				
Without children	61/266 (22.90)	0.580	1.164	0.679-1.998
With children	23/113 (20.40)			
Localities				
Birtouta	15/42 (35.70)	0.025	2.158	1.088-4.278
Bouzareah	12/43 (27.90)	0.336	1.419	0.694-2.903
Dar El beida	10/39 (25.60)	0.581	1.240	0.578-2.660
El Harrach	11/42 (26.20)	0.505	1.283	0.615-2.676
Hussein Dey	06/21 (28.60)	0.467	1.436	0.539-3.824

**2. Consumption habits**				

Consumption place				
Outside of home	25/57 (30.50)	0.040	1.769	1.021-3.066
At home	59/238 (19.90)			
Consumption period				
Summer season	17/70 (24.30)	0.636	1.159	0.630-2.131
Out of summer season	67/309 (21.70)			
Consumption moment				
At lunch	31/117 (26.50)	0.175	1.421	0.854-2.366
At dinner	53/262 (20.20)			

**3. Storage conditions**				

Consumption <2h				
Non	8/24 (25.00)	0.686	1.189	0.513-2.752
Oui	76/271 (21.90)			
Sausages freezing				
Non	61/264 (23.10)	0.503	1.202	0.701-2.061
Oui	23/115 (20.00)			
Keep in the fridge				
Oui	53/168 (24.00)	0.313	1.292	0.784-2.130
Non	31/127 (19.60)			

OR=Odds ratio, CI=Confidence interval

Regarding the demographic characteristics, our results showed five (11) risk factors and found that respondents between the ages of 18 and 40 years (22.60% OR=1.194; [0.571-2.499]), males (25.70%; OR=1.321; [0.785-2.224]), consumers living with their families (23.50%; OR=1.624; [0.810-3.257]) and consumers that had children (22.90%; OR=1.164; [0.679-1.998]) were more affected and more likely to get foodborne diseases after sausage consumption. Thus, the departments Birtouta (35.70%; OR=2.158; p=0.025), Bouzareah (27.90%; OR=1.419), Dar El Beida (25.60%; OR=1.240), El Harrach (26.20%; OR=1.283), and Hussein Dey (28.60%; OR=1.436) had more sick consumers.

Similarly, for the consumption habits, consumers who ate sausages outside of the house (p=0.040; 30.50%), during the summer season (24.30%) or at lunch (26.50%) were found to be risk factors with OR=1.769; (1.021-3.066), OR=1.159; (0.630-2.131); and OR=1.421; (0.854-2.366), respectively. In addition, length of sausage transport (exceeding 2 h) (25%) and sausage stored at room temperature (23.10%; n=61/264); or frozen (24%) were the three risk factors found for the storage conditions category, with; OR=1.189 (0.513-2.752), OR=1.202 (0.701-2.061), and OR=1.292 (0.784-2.130), respectively.

## Discussion

### Bacteriological study

Meat and meat products are the most common foodstuffs in the world, exposing consumers to the risks of *S. aureus* outbreaks with serious economic consequences [15,22]. Although many researchers have previously reported the presence of *S. aureus* in various foods, there is a lack of scientific publications about sausages in Algeria and around the world. To the best of our knowledge, our study is the first epidemiological survey about artisanal sausage (Merguez) in Algeria.

In the present investigation, 25.22% of the evaluated sausages were affected by *S. aureus*. Our results were supported by a sausage study in Saudi Arabia (with 30%) [24] but were lower than that found in Morocco (50%) [14], and the United States of America (42.30%) [13], and higher than that found in Italy (11.77%) [29]. Furthermore, our findings agreed with many meat reports in Algeria (29.40%) [20], Greece (24.50%) [28], Italy (29.41%) [41], Iran (26.31%) [21], and Egypt (23.10%) [42].

For dairy product studies, in Algeria, similar results were reported, as described by [20,43,44]. In contrast, the prevalence obtained was higher than that found in Egypt (12%) [45], (16.60%) [23], Nigeria (9.15%) [46], China (1.8%) [22], Greece (18%) [27], Spain (7%) [26], and Italy (9.79%) [47] (11.77%) [29], with the highest prevalence registered in Georgia [12] and Turkey [48] with (63%) and (42.50%), respectively.

In our study, the overall mean of *S. aureus* contamination was 5.26±0.45 log CFU/g, with the highest contamination level observed in El Harrach (6.06±0.20 log CFU/g), which was characterized by the worst sausage quality. Our results are higher than those described by Ed-Dra *et al*. [14] and Cohen *et al*. [4], with 3.82±0.84 log CFU/g and 2.1±0.41 log CFU/g, respectively. Our results showed that the *S. aureus* count was superior to the maximum tolerable microbiological limit for raw sausages according to the microbiological criteria regarding sausage [37], especially because a contamination level that exceeds 10^5^ *S. aureus* g^−1^ (5.70 log CFU/g) is considered capable of producing a Staphylococcal food poisoning outbreak [49], as was the case for sausage samples from El Harrach, Rouiba, and Bouzareah.

A wide range of *S. aureus* prevalence and contamination levels in sausage were found in this study, indicating the variation in *S. aureus* among the ten (10) departments of Algiers that were studied, with the highest prevalence found in Beraki (68%), Cheraga (44%), and El Harrach (43.75%), which were also the same cities with the worst sausage quality. The different prevalence might be attributed to the geographical location because El Harrach and Beraki represent popular regions with high agglomeration areas and the meat supply departments for the entire capital having the two largest slaughterhouses of cattle and sheep. Nevertheless, the source of *S. aureus* contamination could be multifactorial and differ greatly between countries and even between cities in the same country, especially in the underdeveloped world.

The presence of *S. aureu*s in artisanal sausage (Merguez) may indicate contamination with multiple origins. As hypotheses, we can advance possible contamination of animal origin, a failure in hygiene or recontamination due to insufficient hygienic and sanitary practices [16,50,51] and to the increased number of processes the sausage has been subjected to [27] and could be assigned to the quality of manufacturing practices [34]. Several conditions attest the poor hygienic quality of raw sausage upstream of poor meat quality, possibly due to carcass contamination with intestinal contents during slaughtering and/or the influence of pre-slaughter stress, season, animal density, duration of transport [52], and slaughter-house sanitation [53]. Thus, uncontrolled processing, storage, handling, poor personal hygiene, and sanitary conditions in food industries [34,54] are all factors that can contribute to poor meat quality.

The present study showed that 83.33% of *S. aureus* exhibited resistance to at least one of the antibiotics tested. Moreover, 33% were resistant to three or more antibiotics (multidrug-resistant). Wang *et al*. [22] and Chaalal *et al*. [20] reported similar percentages, 39.10% and 33.30%, respectively; however, high levels of multidrug-resistant isolates were registered in Greece (59.30%) [27] and Morocco (69.84%) [14]. In contrast, a lower rate was found in beef products in the USA [13,25] and dairy milk in Algeria [43].

Our antimicrobial analysis showed that the highest resistance among all antibiotics tested was against tetracycline (58%), followed by oxacillin (36%), fosfomycin (33%), penicillin (25%), and erythromycin (23%). The resistance to tetracycline remains the most common, with a high prevalence, which is in concord with several earlier reports from sausage [14] and different meat products [16,22,23,25,43,55,56]. In contrast, Aydin *et al*. [30] found 8.33% in Turkey sausage; low rates were also recorded in meat and meat products in Italy (25%) [41], and Turkey (22.20%) [48], and Algerian cow milk [43].

For the oxacillin-resistant strain, our findings were similar to those registered in Italy [41] and Algeria [20]. However, sausage studies established by Ed-Dra *et al*. [14] and Aydin *et al*. [30] registered no oxacillin-resistant strains, similar to a result from meat studies by Arslan and Ozdemir [48]. Other researchers have found very low resistance to other antibiotics, such as Wang *et al*. [22], Ge *et al*. [25], and Pu *et al*. [55]. Despite the 25% of strains resistant to penicillin in our study, which is nearly similar to the rate found in sausages in Morocco [14] and in Italian meat [41], earlier studies exhibited a high resistance approaching 90% [22,23,25,30,48,56].

For erythromycin resistance, our results were similar to many studies [20,23,41,55]. Notably, Sergelidis *et al*. [27] and Wang *et al*. [22] found highly resistant strains. On the other hand, low resistance rates of ofloxacin (19%), levomycin (14%), rifampicin (11%), gentamycin (2%), SXT (4%), and cefoxitin (5%) were recorded in our study, which was similar to several meat product studies [22,23,25,27,30,43,50,57] and even Algerian raw milk [43].

In the case of chloramphenicol (10%), resistance is exhibited at more or less lower rates in the different studies, even though, at least for Algeria, treatment by chloramphenicol is banned [57]. Therefore, finding resistance rates may lead to outlawed use. Fortunately, no resistance to vancomycin, teicomycin, or kanamycin was recorded, similar to rates advanced by several researchers, except for kanamycin, where most research showed the beginning of resistance at 6.35% and 8.33% in sausages [14,30]; however, 30% and 32.6% were registered in Egypt [23] and Algeria [20], respectively. In contrast, among the 84 *S. aureus* strains, MAR index analysis demonstrated the presence of 39 different phenotypic profiles ranging from 0 to 0.63, with 20 *S. aureus* profiles belonging to MAR >0.2.

In the present investigation, the results found more resistance against tetracycline, oxacillin, and penicillin and common resistance among *S. aureus* from meat products [12,40] to erythromycin compared to the other antibiotics because of their extensive use as antimicrobial agents against staphylococcal infections, indicating a correlation between antibiotic use and antimicrobial resistance. Tetracycline is a broad-spectrum antimicrobial agent [40] and is used in intensive livestock production, often anarchically, for therapeutic [10,58] and prophylactic purposes or as a growth promoter [59]. However, the irrational use of antibiotics as growth-enhancer drugs has been banned in various countries [60]. Furthermore, the high propagation of antimicrobial resistance in *S. aureus* could be explained by the inappropriate prescription practices of antibiotics used in the animal world [60].

### Epidemiological survey

Foodborne infections are major health concerns in developing countries, including Algeria. Information on consumer habits and their susceptibility to cause outbreaks helps researchers and authorities develop appropriate strategies in terms of prevention, control, and monitoring. The findings of our survey demonstrate the highest consumption frequency of sausage (n=379, 86.14%) among meat consumers, and 84 of the 379 surveyed (22.16%) revealed having had food poisoning after sausage consumption, being more affected and thus more likely to get foodborne diseases after sausage consumption.

For the demographic characteristic risk factors, other studies [61,62] have found similar results in meat consumption among adults and single people; however, males were more likely to be consumers, which is associated with masculinity and power according to Rozin *et al*. [63]. A possible explanation for the differences between males and females may be attributed to the sampling method related to the mode of purchasing in Algeria, dictating that most of the time, the Algerian housewife buys the needs for the home since the man is often at work and returns late. For the purchase locations, the varied places could be a risk factor. Based on our previous study, either the place of purchase and/or the place of consumption could be the cause of foodborne illness. In addition, participants eating during the summer season, outside of the house and at lunch were the three risk factors found in our survey with regard to consumption habits. Similar results were reported by Wu *et al*. [64] with 65.70% and by Kadariya *et al*. [15] with 44% of outbreaks occurring outside of the home. This may be due either to the fact that most Algerians are at work during the day and have no choice other than eating out, which is consistent with lunchtime, and the lack of awareness consumers has regarding insufficient hygienic and sanitary conditions [15,52,65], since handling during preparation is considered the main source of food contamination [16].

Furthermore, our investigation revealed three other risk factors interpreted as poor storage habits including the length of transport (exceeding 2 h after purchase); the sausage storage methods at home, before and during consumption; and the fact that consumers freeze the sausage. Similar results were reported by various studies [15,66,67]. According to Kadariya *et al*. [15], several errors in food preparation are the most common contributing factor (93%) in foodborne diseases, as well as storage conditions (39%) and prolonged exposure of foods at ambient temperature (58%), supporting the notion that there are limited health consciousness and poor knowledge of good hygienic practices among Algerian consumers. Therefore, consumers need to be aware that traditional sausages, widely consumed in Algeria, are potential risks and could cause foodborne diseases if handled inappropriately at home. Thus, more information about hygiene rules and preparation is necessary for the general population and, more urgently, for groups at risk [64].

## Conclusion

Our comprehensive report highlights a high prevalence of *S. aureus* sausage contamination found in some departments of Algeria, high multidrug resistance identified in isolates, and amount of *S. aureus* that is above the limit established by law and at levels compatible with the production of enterotoxins, which revealed the potential risk of artisanal sausage to public health in Algeria. Therefore, to reduce resistant strains, monitoring antibiotics and developing new treatment strategies against staphylococcal infections should be established, especially those that are still widely used in Algeria for human therapy because of their low cost and availability. Furthermore, it is necessary to improve education regarding Algerian sausage by changing certain consumption habits related to the risk factors found. Finally, it can be concluded that authority interventions should be designed to prevent *S. aureus* contamination during pre-slaughter, post-slaughter and at butcheries producing sausage, targeting adherence to either good hygiene practices and/or HACCP system. From this perspective, studies might be performed to characterize *Staphylococcus* spp. and *S. aureus* to investigate their virulence factors.

## Authors’ Contributions

AH conducted the studies, experimental procedures, and analysis. KA participated in the design of the studies. MMH and MG participated in sample collection. AH and MFD were responsible for antibiogram analysis. AH and SZ were carried out the statistical analysis and interpretation. The manuscript was drafted by AH and reviewed by AH, SZ, and KA. All authors read and approved the final manuscript.
